# On the role of gallbladder emptying and incretin hormones for nutrient-mediated TSH suppression in patients with type 2 diabetes

**DOI:** 10.1530/EC-14-0088

**Published:** 2014-10-18

**Authors:** David P Sonne, Asger Lund, Jens Faber, Jens J Holst, Tina Vilsbøll, Filip K Knop

**Affiliations:** 1 Department of Medicine, Center for Diabetes Research, Gentofte Hospital, University of Copenhagen, Niels Andersens Vej 65DK-2900, Hellerup, Denmark; 2 Department of Biomedical Sciences, Faculty of Health and Medical Sciences, The NNF Center for Basic Metabolic Research, University of Copenhagen, Copenhagen, Denmark; 3 Department of Endocrinology, Herlev Hospital, University of Copenhagen, Herlev, Denmark

**Keywords:** bile acids, gallbladder, glucagon-like peptide 1, GLP1, thyroid, thyroid-stimulating hormone, TSH, TGR5, type 2 diabetes

## Abstract

Bile acids are possible candidate agents in newly identified pathways through which energy expenditure may be regulated. Preclinical studies suggest that bile acids activate the enzyme type 2 iodothyronine deiodinase, which deiodinates thyroxine (T_4_) to the biologically active triiodothyronine (T_3_). We aimed to evaluate the influence of bile acid exposure and incretin hormones on thyroid function parameters in patients with type 2 diabetes. Thyroid-stimulating hormone (TSH) and thyroid hormones (total T_3_ and free T_4_) were measured in plasma from two human studies: i) 75 g-oral glucose tolerance test (OGTT) and three isocaloric (500 kcal) and isovolaemic (350 ml) liquid meals with increasing fat content with concomitant ultrasonographic evaluation of gallbladder emptying in 15 patients with type 2 diabetes and 15 healthy age, gender and BMI-matched controls (meal-study) and ii) 50 g-OGTT and isoglycaemic intravenous glucose infusions (IIGI) alone or in combination with glucose-dependent insulinotropic polypeptide (GIP), glucagon-like peptide 1 (GLP1) and/or GLP2, in ten patients with type 2 diabetes (IIGI-study). In both studies, TSH levels declined (*P*<0.01) similarly following all meal and infusion stimuli. T_3_ and T_4_ concentrations did not change in response to any of the applied stimuli. TSH levels declined independently of the degree of gallbladder emptying (meal-study), route of nutrient administration and infusion of gut hormones. In conclusion, intestinal bile flow and i.v. infusions of the gut hormones, GIP, GLP1 and/or GLP2, do not seem to affect thyroid function parameters. Thus, the presence of a ‘gut–thyroid–pituitary’ axis seems questionable.

## Introduction

Bile acids are water soluble, amphipathic molecules synthesised in the liver from cholesterol. Upon meal ingestion, the gallbladder contracts, whereby bile acids from the liver and highly concentrated bile acids from the gallbladder are released into to the intestinal lumen. Here they interact with dietary lipids, lipid-soluble vitamins and cholesterol, forming mixed micelles, and thereby facilitating the transport and uptake of these molecules (1). Nowadays, bile acids are no longer labelled as detergents necessary for lipid digestion and absorption, but are increasingly recognised as metabolic integrators, capable of regulating glucose homeostasis, lipid metabolism and energy expenditure through nuclear receptors and the G protein-coupled receptor TGR5 (2, 3, 4). A study by Watanabe *et al*. (4) carried out in mice showed that cholic acid supplementation augmented energy expenditure in brown adipose tissue, caused weight reduction and improved insulin sensitivity. The effects were mediated through TGR5-induced intracellular cAMP formation and activation of the intracellular type 2 iodothyronine deiodinase (D2), which converts thyroxine (T_4_) into the active thyroid hormone triiodothyronine (T_3_).

Up to date, human studies have yielded conflicting results regarding the association between circulating bile acids and thyroid function parameters. Patti *et al*. (5) have found that bile acids in serum correlated inversely with thyroid-stimulating hormone (TSH) in non-diabetic patients who had undergone Roux-en-Y gastric bypass surgery, and recent reports have demonstrated similar associations of bile acids with TSH in type 2 diabetes (6, 7, 8). In contrast, Brufau *et al*. (9) could not demonstrate any effect of bile acids on energy expenditure in patients with type 2 diabetes. Recently, Ockenga *et al*. (8) showed that TSH concentrations decreased post-prandially in both patients with liver cirrhosis and healthy controls, and the result furthermore indicated that post-prandial bile acids are capable of increasing D2 activity, which in turn converts T_4_ into T_3_ presumably suppressing TSH upon meal intake. Such inhibitory effect of nutrient ingestion on TSH secretion in humans has been sparsely reported in the literature (10, 11). As meal-stimulated gallbladder emptying leads to prompt elevations of plasma bile acids concentrations (12, 13), we speculated whether various degrees of gallbladder emptying – induced by meals with a wide range of fat content – would modulate the thyroid hormone axis accordingly in both type 2 diabetes patients and controls. Moreover, in a second study in type 2 diabetes patients, using oral glucose tolerance test (OGTT) and isoglycaemic intravenous glucose infusions (IIGI), we analysed i) whether i.v. glucose *per se* could affect thyroid function parameters and ii) whether any changes could be elicited by infusion of the gastrointestinal hormones glucose-dependent insulinotropic polypeptide (GIP), glucagon-like peptide 1 (GLP1) and GLP2, which are known to be secreted after nutrient ingestion.

## Subjects and methods

### Meal-study

Detailed description of the experimental procedures and subjects was provided previously (14). In short, measurement of TSH, total T_3_ and free T_4_ was carried out with chemiluminescence immunoassays in the plasma from 15 patients with type 2 diabetes (mean duration of diabetes, 7.5 years (range 6–20); age, 59.4±9.6 years (mean±s.d.); BMI, 28.0±2.2 kg/m^2^ and HbA1c, 7.5±1.4%) and 15 healthy age, gender and BMI-matched control subjects (age, 59.7±10.0 years; BMI, 27.9±2.0 kg/m^2^ and HbA1c, 5.2±0.2%) undergoing four separate ‘meal’ tests: a 75 g-OGTT and three isocaloric (500 kcal) and isovolaemic (350 ml) liquid meals (low fat: 2.5 g fat, 107 g carbohydrate and 13 g protein; medium fat: 10 g fat, 93 g carbohydrate and 11 g protein and high fat: 40 g fat, 32 g carbohydrate and 3 g protein). Four type 2 diabetes patients were treated with diet alone, eight were also treated with metformin and three with sulphonylurea (any oral antidiabetic therapy was omitted for a period of no less than a week before each study day). Gallbladder emptying was evaluated by calculating gallbladder ejection fraction using ultrasound measurements as previously described (14).

### IIGI-study

Detailed experimental procedures and patient characteristics were reported previously (15). In short, measurement of TSH, total T_3_ and free T_4_ was carried out with chemiluminescence immunoassays in plasma from ten patients with type 2 diabetes (mean duration of diabetes, 4.8 years (range 0.5–14); age, 50.8±10.7 years (mean±s.d.); BMI, 33.2±4.9 kg/m^2^ and HbA1c, 6.5±0.7%) undergoing six separate test days: 50 g-OGTT, IIGI+saline (placebo), IIGI+GIP, IIGI+GLP1, IIGI+GLP2 and IIGI+GIP+GLP1+GLP2 (saline and peptides were infused intravenously). Nine patients were treated with metformin alone and one with metformin and sulphonylurea in combination (any oral antidiabetic therapy was omitted for a period of not less than a week before each study day).

All participants gave their informed consent to the studies, which were performed in accordance with the Declaration of Helsinki and Danish legislation.

### Calculations and statistical analyses

The results are reported as means with 95% CIs unless otherwise stated. For evaluation of TSH suppression within groups, repeated measures two-way ANOVA with Dunnet's *post hoc* test was used (mean baseline concentrations were chosen as control mean). For between-group comparison of TSH responses, area under the curve (AUC) was calculated by applying the trapezoid rule for TSH. For analysis of variations and differences between AUC values, repeated measures ANOVA with Sidak's *post hoc* test was used. The data were transformed logarithmically (log10) to approximate a Gaussian distribution. A two-sided *P*<0.05 was used to indicate significant differences.

## Results

### Meal-study

**Figure 1:**
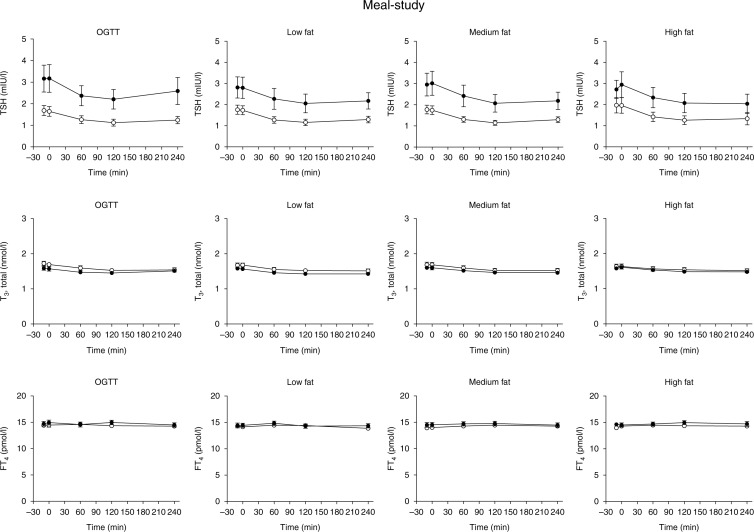
Meal-study. Plasma levels of thyroid-stimulating hormone (TSH), thyroxine (T_4_) and thyroid hormone triiodothyronine (T_3_) during a 75 g-oral glucose tolerance test (OGTT) and three isocaloric (500 kcal) and isovolaemic (350 ml) liquid meals with low fat, medium fat and high fat, in healthy control subjects (*n*=15, closed symbols) and patients with type 2 diabetes (*n*=15, open symbols). Mean±s.e.m. values are shown.

As previously reported, gallbladder ejection fraction increased similarly in the two groups with increasing meal fat content (with similar ejection fraction following the medium fat meal and the high fat meal nevertheless) (14). Baseline TSH levels were within normal range (0.3–4.0 mIU/l) in all participants. As shown in Fig. 1, AUC levels and basal TSH concentrations were higher in controls vs type 2 diabetes patients during oral glucose (*P*<0.05, Fig. 1); a tendency to this was also seen during the meal stimuli (*P*=0.05–0.09). TSH levels declined significantly after the applied stimuli in both groups with maximum changes from baseline amounting to −0.54 UI/l (−0.75, −0.33) (OGTT), −0.59 (−0.78, −0.41) (low fat), −0.62 (−0.84, −0.39) (medium fat) and −0.70 (−1.10, −0.34) (high fat) in the type 2 diabetic group and −0.96 (−1.40, −0.53) (OGTT), −0.75 (−1.06, −0.45) (low fat), −0.92 (−1.33, −0.51) (medium fat) and −0.76 (−0.99, −0.52) (high fat) in controls, with no statistical significant differences within or between groups. Neither, incremental AUC values differed within or between groups. The concentrations of T_3_ and T_4_, respectively, were similar in the two groups and did not change after any of the oral stimuli.

### IIGI-study

**Figure 2:**
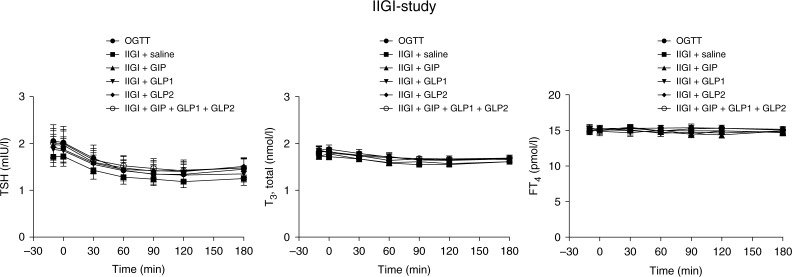
IIGI-study. Plasma levels of thyroid-stimulating hormone (TSH), thyroxine (T_4_) and thyroid hormone triiodothyronine (T_3_) during 50 g-oral glucose tolerance test (OGTT) and isoglycaemic intravenous glucose infusions (IIGI) with concomitant infusions of saline, glucose-dependent insulinotropic polypeptide (GIP), glucagon-like peptide 1 (GLP1), GLP2 or a combination of the three hormones in patients with type 2 diabetes (*n*=10). Mean±s.e.m. values are shown.

TSH declined similarly on all study days following oral glucose intake and all of the i.v. stimuli respectively. Accordingly, maximum changes from baseline amounted to −0.76 (−1.15, −0.37) (OGTT), −0.56 (−0.79, −0.33) (IIGI+saline), −0.64 (−0.89, −0.38) (IIGI+GIP), −0.57 (−0.82, −0.32) (IIGI+GLP1), −0.55 (−0.82, −0.28) (IIGI+GLP2) and −0.63 (−0.92, −0.34) (IIGI+GIP+GLP1+GLP2), with no statistical significant differences between the different study days. Also, values of AUCs were similar during the different study days (Fig. 2). The concentrations of T_3_ and T_4_, respectively, were comparable along the entire time course on all study days. Neither suppression nor elevation of T_3_ or T_4_ was demonstrated.

## Discussion

This study demonstrates a similar degree of TSH suppression in type 2 diabetes patients and healthy controls to oral glucose and isocaloric and isovolaemic meals with increasing fat content (giving rise to different gallbladder emptying rates), and suggests that the oral route of nutrient administration and subsequent release of the gut hormones, GIP, GLP1 and GLP2 do not seem to play a major role in post-absorptive TSH suppression. To our knowledge, no previous study has compared thyroid function parameters following both OGTT and various meal tests (isocaloric and isovolaemic) affecting gallbladder emptying differentially in type 2 diabetes patients and matched healthy controls, or thyroid function parameters after oral and i.v. glucose in type 2 diabetes.

In both studies, basal and stimulated TSH concentrations were within normal range, but tended to be lower in type 2 diabetes patients vs controls (meal-study). This contrasts to the common observation of subclinical hypothyroidism in type 2 diabetes patients (16, 17). Interestingly, recent data indicates that metformin may suppress TSH concentrations in type 2 diabetes patients, thus providing a plausible explanation of the low(er) TSH levels in our type 2 diabetic patients (18). However, contrasting results have also been reported (19). In spite of the observed TSH suppression, concentrations of T_3_ and T_4_ did not exhibit post-prandial changes and were similar in type 2 diabetes patients vs controls. This is supported by a previous study in hypothyroid patients, showing that T_3_ and T_4_ were relatively insensitive to small alterations in T_4_ (administered daily) compared with TSH, which was very sensitive to fine adjustments of T_4_ dosage (20). The notion of oral glucose eliciting a similar degree of TSH suppression as compared with IIGI suggests that gastrointestinal factors are less likely to play a role in nutrient-induced TSH suppression. Supporting this notion, possible candidate ‘gut factors’, such as GIP, GLP1 and GLP2, infused intravenously during IIGI neither altered the suppression pattern of TSH or thyroid hormones. By contrast, a previous study indicated that peripheral T_3_ formation was stimulated by oral, but not by i.v., glucose suggesting that the intestine or liver might involve in the regulation of T_3_ formation (21).

As plasma bile acid concentrations reflect intestinal bile acid absorption, as the fractional hepatic uptake of bile acids is constant (13), the degree of gallbladder contraction can be considered an ‘indirect’ measure of the amount of bile acids that reaches the systemic circulation (‘spill-over’ from liver). On the basis of this assumption, our results do not support a role for post-prandial bile acids in modulating bile acid–TGR5-D2 activation in peripheral tissues (skeletal muscle and brown adipose tissue). However, we acknowledge that the study design does not allow drawing causal inferences in this regard. Indeed, gallbladder emptying and even bile acids provide only surrogate information of the complexity of the enterohepatic circulation of bile acids even though both are positively correlated with portal venous concentrations (22).

A prompt inhibitory effect of nutrient ingestion on TSH secretion in humans has been sparsely reported in the literature (8, 10, 11) and the mechanism underlying the suppression of TSH after ingestion of meals or glucose remains to be established. In fact, TSH concentrations are generally believed to decrease following long-term fasting and increase upon refeeding (23, 24, 25). Considering the role of gallbladder emptying and systemic bile acid fluctuations, bile acid–TGR5 signalling might, as hypothesised by Ockenga *et al*., acutely activate D2 in pituitary thyrotropes, which are known to express D2 (26) and possibly TGR5 (8), resulting in feedback on TSH upon meal challenge. Importantly, such activation of D2 may also be potentiated from the skeletal muscle where D2 activity is sufficiently high that it can provide significant quantities of circulating T_3_ (27). Intriguingly, in rodents TGR5 expression has been described in the paraventricular nucleus and in the supraoptic nucleus of the hypothalamus, suggesting that bile acid–TGR5 signalling may regulate the thyroid axis at the level of thyrotropin-releasing hormone (8, 28). In support of this, Ockenga *et al*. (8) demonstrated an association between bile acid and TSH concentrations after a nutrient challenge (0–60 min) and found a close correlation between meal-stimulated bile acid levels and energy expenditure after 60 min (most evident in patients with liver cirrhosis). However, D2 activation might also simply be enhanced by nutrients – especially glucose (29, 30) – reaching the systemic circulation either via the oral route or during i.v. stimulation. Interestingly, this is corroborated well in another human study, which showed that 62 h of fasting reduced *D2* mRNA expression in skeletal muscle, whereas 5 h of insulin infusion increased *D2* mRNA expression (at 62 h) (31). However, D2 activities were very low and were not influenced by hypothyroidism, fasting or insulin.

Another explanation for the acute post-prandial TSH decline observed in this study could be somatostatin being released concomitantly from the intestine and/or hypothalamus upon nutrient stimulation (10, 11, 32). However, the physiological role of somatostatin for the inhibition of TSH secretion in man has been questioned (33). Accordingly, our observation of i.v. glucose infusions suppressing TSH levels to similar levels as observed during oral glucose argues against gut-derived somatostatin playing a role in post-prandial inhibition of TSH release. In contrast, hypothalamic somatostatin secretion could have been stimulated during both oral and i.v. glucose and thereby contribute to the observed TSH suppression patterns (11). Also, as GIP and GLP1 increase somatostatin secretion, at least in animals (34), suppression of TSH during incretin infusion might have been augmented, which, however, was not demonstrated in this study. Moreover, hypoglycaemia, which is known to suppress somatostatin levels, has also been shown to reduce TSH concentrations (35), indicating that other factors apart from somatostatin are responsible for TSH suppression under normal physiological circumstances.

Finally, TSH concentrations display pronounced circadian changes – typically with nocturnal increases and daytime nadirs, which might also offer an explanation for the decline observed after all stimuli (36). However, a recent study in 475 000 outpatients has indicated that TSH concentrations are constant from 0600 to 1900 h (37). Accordingly, TSH baseline concentrations in this study were similar, and suppression was noticeable only after the nutrient stimuli were applied. Nonetheless, it remains an obvious limitation to this study, as well as the study by Ockenga *et al*. (8), that TSH concentrations were not evaluated following non-nutrient stimuli (negative control).

In conclusion, patients with type 2 diabetes and healthy controls display significant post-prandial TSH suppression to a series of meal-related stimuli, but independently of the degree of gallbladder emptying and of the gut hormones GIP, GLP1 and GLP2. Obviously, further studies are warranted to clarify whether bile acids affect energy expenditure and diet-induced thermogenesis (via brown adipose tissue) in humans, but the present findings do not support the presence of a ‘gut–thyroid–pituitary’ axis.

## Author contribution statement

D P Sonne: study design, clinical experiments (meal-study), data research, statistical analysis and drafting of manuscript; A Lund: clinical experiments (IIGI-study) and review and editing of the manuscript; J Faber, J J Holst and T Vilsbøll: review and editing of the manuscript and F K Knop: study design, and review and editing of the manuscript. All authors have read and approved the final version of the manuscript.
